# Fe-MOF Catalytic Nanoarchitectonic toward Electrochemical Ammonia Production

**DOI:** 10.1021/acsami.3c12822

**Published:** 2023-10-02

**Authors:** Akshay Kumar K. Padinjareveetil, Juan V. Perales-Rondon, Dagmar Zaoralová, Michal Otyepka, Osamah Alduhaish, Martin Pumera

**Affiliations:** † Future Energy and Innovation Laboratory, Central European Institute of Technology, Brno University of Technology, Purkyňova 123, Brno 612 00, Czech Republic; ‡ IT4Innovations, VŠB − Technical University of Ostrava, Ostrava-Poruba 708 00, Czech Republic; § Regional Centre of Advanced Technologies and Materials, Czech Advanced Technology and Research Institute (CATRIN), Palacký University Olomouc, Olomouc 783 71, Czech Republic; ∥ Chemistry Department, College of Science, King Saud University, P.O. Box 2455, Riyadh 11451, Saudi Arabia; ⊥ Faculty of Electrical Engineering and Computer Science, VSB - Technical University of Ostrava, 17. listopadu 2172/15, Ostrava 708 00, Czech Republic; # Department of Paediatrics and Inherited Metabolic Disorders, First Faculty of Medicine, Charles University Prague, KeKarlovu 2, Prague 128 08, Czech Republic; ∇ Department of Medical Research, China Medical University Hospital, China Medical University, No. 91 Hsueh-Shih Road, Taichung 40402, Taiwan

**Keywords:** metal−organic framework, PCN-250-Fe_3_, ammonia synthesis, thermal activation, electrochemical nitrate reduction, electrocatalysts

## Abstract

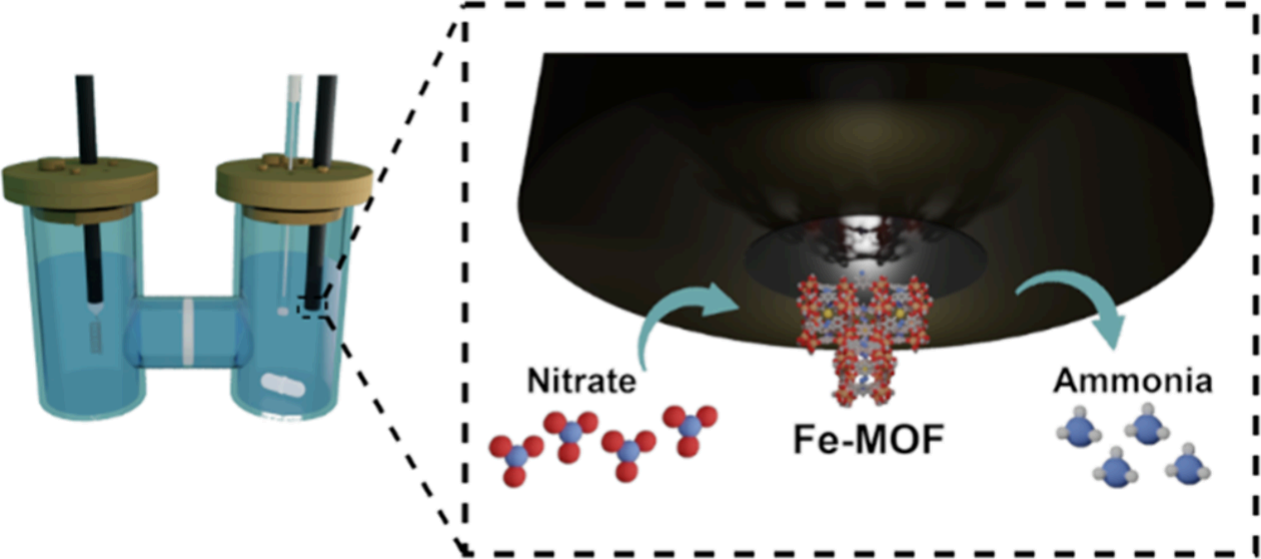

Electrochemical reduction of nitrate into ammonia has lately been identified as one among the promising solutions to address the challenges triggered by the growing global energy demand. Exploring newer electrocatalyst materials is vital to make this process effective and feasible. Recently, metal–organic framework (MOF)-based catalysts are being well investigated for electrocatalytic ammonia synthesis, accounting for their enhanced structural and compositional integrity during catalytic reduction reactions. In this study, we investigate the ability of the PCN-250-Fe_3_ MOF toward ammonia production in its pristine and activated forms. The activated MOF catalyst delivered a faradaic efficiency of about 90% at −1 V vs RHE and a yield rate of 2.5 × 10^–4^ mol cm^–2^ h^–1^, while the pristine catalyst delivered a 60% faradaic efficiency at the same potential. Theoretical studies further provide insights into the nitrate reduction reaction mechanism catalyzed by the PCN-250-Fe_3_ MOF catalyst. In short, simpler and cost-effective strategies such as pretreatment of electrocatalysts have an upper hand in aggravating the intrinsic material properties, for catalytic applications, when compared to conventional material modification approaches.

## 1 Introduction

The exponential growth in the global population has led to a significant decline in the availability of fossil fuels and increased energy demands, especially in a low-carbon economy. Exploring clean, secure, and renewable energy sources therefore has become a matter of serious concern for the scientific community across the world.^1−5^ Studies have shown ammonia to be a potential carbon-free candidate to mitigate the growing energy demand, owing to its high gravimetric energy density (3 kWh kg^–1^) and high hydrogen capacity (17.65 wt %), facilitating ease in storage and transportation, along with clean emissions.^6−9^ Along with being a green hydrogen-rich fuel, ammonia is also fundamental to the production of fertilizers in modern agricultural sectors and finds diverse applications in fields such as pharmaceuticals, textiles, refrigeration, etc. The conventional strategy employed for the large-scale production of ammonia is the Haber–Bosch process,^10,11^ where both nitrogen and hydrogen are subjected to high-temperature (400–500 °C) and high-pressure (150–300 atm) conditions in the presence of a heterogeneous iron (Fe)-based catalyst. However, being an energy intensive process, involving large emissions of carbon dioxide (CO_2_), there have been rigorous attempts in finding alternatives to this method.^12−16^ Electrochemical ammonia synthesis has emerged as a suitable technique for ammonia production, with nitrogen reduction reaction being the most extended and studied method.^17−20^ Although the process has evolved with time, yet limitations such as low solubility, high bond dissociation energy of N≡N (941 kJ mol^–1^), low selectivity at high current densities, modest faradaic efficiency (FE), low yield rate, competitive hydrogen evolution reaction (HER), and sluggish kinetic hamper its frequent use.^21−23^ The limitations therefore invite further improvements and newer techniques for enabling large-scale production under mild conditions.

Alternative techniques such as nitrate reduction into ammonia (NRA) have gained momentum for ammonia synthesis, addressing several limitations faced by the aforementioned techniques.^24−26^ Recognizing the extensive presence of nitrate ions, in the environment, especially as a pollutant in waters, it has been considered as a perfect alternative nitrogen source for ammonia synthesis as well.^27,28^ Designing a strategy to eradicate a water pollutant like nitrate, which also brings a serious threat to human health, represents an advantage in terms of both energy production and addressing environmental issues. Thus, the nitrate electroreduction can be considered as an eco-friendly and efficient approach of converting aqueous waste nitrate into ammonia under favorable operating conditions.^29^ The conversion of nitrate to ammonia involves an eight-electron transfer that proceeds through multiple reaction pathways at a definite potential region^30,31^ However, side reactions such as HER can also occur in this region and result in the consumption of electrons for hydrogen generation and eventually in decreased FE and selectivity. Thus, the necessity for designing specific electrocatalysts is of utmost importance, which dismisses both N≡N bond formation and competitive HER, and also efficiently reducing nitrate into ammonia is of utmost importance. Electrocatalysts such as transition metals,^31,32^ their oxides,^33^ metal single-atom catalysts,^34^ or alloys^24^ have been studied for ammonia production from nitrate. Tailoring the surface of the electrocatalysts is also another strategy to enhance the properties of the catalyst. For instance, surface modifications of electrocatalysts with negatively charged species are known to suppress the HER interference during the reaction, resulting in enhanced activity and selectivity for NRA applications.^35^ In another study, 2D Ti_3_C_2_T_*x*_ MXene was also used as a suitable substrate to disperse and anchor copper (Cu) over it, resulting in molecular Cu@MXene catalysts.^36^ The catalyst gave around a 94% ammonia selectivity and 90.5% nitrate conversion rate, thereby opening up newer strategies to develop electrocatalysts for NRA.

From the past two decades, metal–organic frameworks (MOFs) have gained significant recognition owing to their porous nature, crystalline structure, tunable functionality, and high surface area within the single entity of the material.^37,38^ The members of this emerging group are synthesized by self-assembling of organic ligands with desired metal centers, having several potential applications.^39^ Previously, MOFs have been well studied for gas storage, separation, energy storage, and multiple other applications. However, only fewer studies have been reported on MOFs for the ammonia production via NRA. In a recent study, a MOF-derived cobalt (Co)-doped Fe/Fe_2_O_3_ catalyst has been reported by Zhang et al. for electrochemical nitrate reduction.^40^ The electronic structure of the Fe d band center was tuned via Co doping, resulting in the modulation of adsorption energy of intermediates and inhibiting hydrogen formation. This Co-doped Fe/Fe_2_O_3_ MOF electrocatalyst resulted in a 99% ammonia selectivity, an FE of 85.2%, and a high nitrate removal capacity of 100.8 mg N/g_cat_ h. In another recent work by Qin et al., Ru_*x*_O_*y*_ clusters anchored on nickel (Ni) MOFs (RuNi-MOFs) were studied for electrocatalytic NRA.^41^ The catalyst achieved an FE of 73% at −1.2 V vs Ag/AgCl for NH_4_^+^ and NH_4_^+^–N yield rates of 274 μg h^–1^ mg_cat_^–1^ at −1.7 V vs Ag/AgCl.

The above works clearly discuss the possibility of engineering MOFs via heteroatom doping and anchoring molecular catalysts for efficient electrocatalytic NRA. However, since this study is still in its infancy, exploring the further possibility of modifying MOFs without any doping or anchoring of active components becomes relevant to understand the real potential of MOFs in this field. For instance, studies on the differences in the intrinsic material property when subjected to thermal activation have never been undertaken before, especially for electrocatalytic applications such as ammonia production. Such observations can be vital, especially in enabling direct enhancement of material toward ammonia synthesis, without any additional and time-consuming techniques such as doping, substitution, functionalization, etc.

In this study, an Fe metal center-based MOF, known as the PCN-250-Fe_3_ MOF, has been examined for electrocatalytic NRA in both its pristine and activated forms. The activated material gave a substantial improvement in FE (∼90% at −1.0 V vs reversible hydrogen electrode (RHE)) when compared to its pristine form. Cyclic stability and MOF stability in solvents with time are also assessed as a part of this study. The thermodynamic feasibility of the PCN-250-Fe_3_ MOF catalyst toward NRA is corroborated by density functional theory (DFT) calculations as well. This study is a breakthrough in NRA using MOF materials, especially in understanding the importance of material pretreatments for catalytic applications.

## 2 Experimental Section

### 2.1 Reagents and Materials

All chemical reagents, such as potassium nitrate (KNO_3_), sodium sulfate (Na_2_SO_4_), hydrochloric acid (HCl), sulfamic acid (H_3_NSO_3_), *p*-aminobenzenesulfonamide (H_2_NC_6_H_4_SO_2_NH_2_), *N*-(1-naphthyl)ethylenediamine dihydrochloride (C_10_H_7_NHCH_2_CH_2_NH_2_·2HCl), phosphoric acid (H_3_PO_4_), sodium hydroxide (NaOH), ammonium chloride (NH_4_Cl), citric acid (HOC(COOH)(CH_2_COOH)_2_), salicylic acid (2-(HO)C_6_H_4_CO_2_H), sodium hypochlorite solution (NaClO, 6–14%), sodium nitroferricyanide (Na_2_[Fe(CN)_5_NO]), and sodium nitrite (NaNO_2_), were used as received from Merck and Sigma-Aldrich Co., Ltd., without further purification. The PCN-250-Fe_3_ MOF also was procured from commercial sources, called framergy. All solutions were prepared by using ultrapure water (18.2 MΩ cm resistivity at 25 °C).

### 2.2 Characterization

The scanning electron microscopy (SEM) images were obtained from a LYRA 3 SEM (TESCAN) and Verios 460L (Thermo Fisher Scientific, USA). The energy-dispersive X-ray spectroscopy (EDS) images were obtained with a Bruker XFlash 5010 detector attached with the LYRA. X-ray photoelectron spectroscopy (XPS) was measured using a Kratos AXIS Supra instrument with monochromatized Al *K*_α_ excitation (1486.7 eV), and the data were analyzed using CasaXPS software. The X-ray diffraction (XRD) measurements were conducted with a diffractometer (SmartLab 3 kW, Rigaku) with a Bragg–Brentano geometry (Cu *K*_α_ radiation; λ = 0.15418 nm) operated at a voltage of 40 kV and a current of 30 mA. The ultraviolet–visible (UV–vis) absorbance spectra from the wavelength range 190 to 900 nm were measured on a double-beam Jasco Co. Model V-750. ^1^H NMR experiments were conducted using a 500 MHz Bruker, ADVANCE NEO 4500 de.

### 2.3 Sample Preparation

The MOF samples were activated using a vacuum oven at 150 °C for 3 h. Further, the MOF samples were measured to obtain a 1 mg·mL^–1^ concentration in 10 mL of distilled water. To obtain an optimal dispersion, the solution was sonicated using an ultrasonic homogenizer probe for 30 min at an amplitude of 70%, 20 s of sonication, and 10 s of resting. To this dispersion, 40 μL of Nafion binder (∼5% in a mixture of lower aliphatic alcohols and water) is added and sonicated well. From the stock solution, 10 μL of sample is drop-casted over the glassy carbon (GC) electrode and dried prior to assembling it in the H cell. Studies on pristine/nonactivated samples were carried out by direct sonication of MOF samples in the similarly ascribed concentration range and further analyzed for NRA.

### 2.4 Electrochemical Measurements

The electrochemical measurements were carried out using an Autolab PGSTAT204 (Metrohm) operated by Nova 2.14 software. The electrolysis experiments were conducted in an H-type electrolytic cell with a frit separation. The PCN-250-Fe_3_ MOF over GC served as the working electrode at the cathodic end of the H cell along with the commercial Ag/AgCl reference electrode, while platinum wire served as the counter electrode at the anodic end. The electrolytic experiments were conducted at multiple potentials (−0.6 to −1.4 V vs RHE), with each experiment carried out for 1 h with a constant magnetic stirring rate (100 rpm). All potentials were recorded against the RHE. The conversion of Ag/AgCl to RHE is carried out via the following equation: *E*_RHE_ = *E*_Ag/AgCl_ + 0.0591 pH + 0.199.

### 2.5 Colorimetric Determination of Ion Concentrations

Quantification of the ion concentration of different products was carried out using well-known colorimetric methods. A UV–vis spectrophotometer was used to detect the concentration of different reagents/products of pre- and post-electrolysis experiments.

### 2.6 Determination of Ammonia

Ammonia (NH_3_) concentration after electrolysis was determined by the well-known indophenol blue method.^34^ Once the electrolytic experiment was performed, an aliquot of the electrolyte was taken out from the cell, and a proper dilution to 600 μL of solution was done. Subsequently, 600 μL of a 3 M NaOH solution containing 10 wt % salicylic acid and 10 wt % sodium citrate was added to the solution. After that, 300 μL of 0.20 M NaClO and 60 μL of 2.0 wt % C_5_FeN_6_Na_2_O (sodium nitroferricyanide) solution was also added to the solution. The resulting solution was allowed to rest for 2 h, after which the UV–vis absorption spectrum was taken. NH_3_ concentration was determined by the formation of the indophenol blue product that was quantified using the absorbance at a wavelength of 655 nm. The corresponding calibration curve was obtained using standard solutions of ammonium chloride.

### 2.7 Determination of Nitrite

A previously reported quantitative protocol was used to carry out nitrite (NO_2_^–^) determination.^42,43^ To do so, a color reagent containing *p*-aminobenzenesulfonamide (0.4 g), *N*-(1-naphthyl)ethylenediamine dihydrochloride (0.02 g), ultrapure water (5 mL), and phosphoric acid (1 mL, ρ = 1.70 g/mL) was prepared. Post electrolysis, a certain volume of electrolyte was taken out from the cell and diluted to 1.5 mL to the detection range. After that, 50 μL of the color reagent was added into the 1.5 mL solution, followed by the addition of 100 μL of phosphoric acid (ρ = 1.70 g/mL), and mixed uniformly. The absorption intensity at a wavelength of 540 nm was recorded after the solution rested for 20 min. The calibration curve concentration–absorbance was carried out by using a series of standard sodium nitrite solutions.

### 2.8 Determination of Ammonia by the ^1^H NMR Quantitative Method and Isotopic Labeling Experiment

Post electrolysis, an aliquot of the electrolyte was collected from the H cell. Concentrated H_2_SO_4_ (250 μL) was added to 5 mL of electrolyte to ensure a high acidic condition that is ideal to be quantified by ^1^H NMR and using maleic acid as an internal standard. The calibration curve was carried out as follows: a series of standard solutions of known concentrations of ^14^NH_4_^+^ (50, 100, 150, 200, and 250 ppm) were prepared in 0.5 M Na_2_SO_4_ + 0.1 M KNO_3_. Next, 5 mL of each solution was mixed with 0.002 g of maleic acid. To carry out the measurement, 500 μL of this solution was placed in an NMR tube, and 50 μL of deuterium oxide (D_2_O) was added to it for the NMR detection. The calibration curve was achieved by using the peak area ratio between ^14^NH_4_^+^ and maleic acid.

### 2.9 Calculation of Different Parameters to Evaluate the Performance of the Electrocatalysts


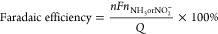
1
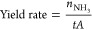
2where *F* is the faradaic constant (96485 C mol^–1^), *n*_NH_3_ or NO_2_^–^_ is the number of mol of NH_3_ or NO_2_^–^, *n* is the number of electrons involved in the electrochemical reaction (8 for NH_3_ and 2 for NO_2_^–^), *t* is the electrolysis time (1 h), *A* is the area of the electrode, and *Q* is the total charge measured during the electrolytic experiment.

### 2.10 Computational Study Details

**Scheme 1:**
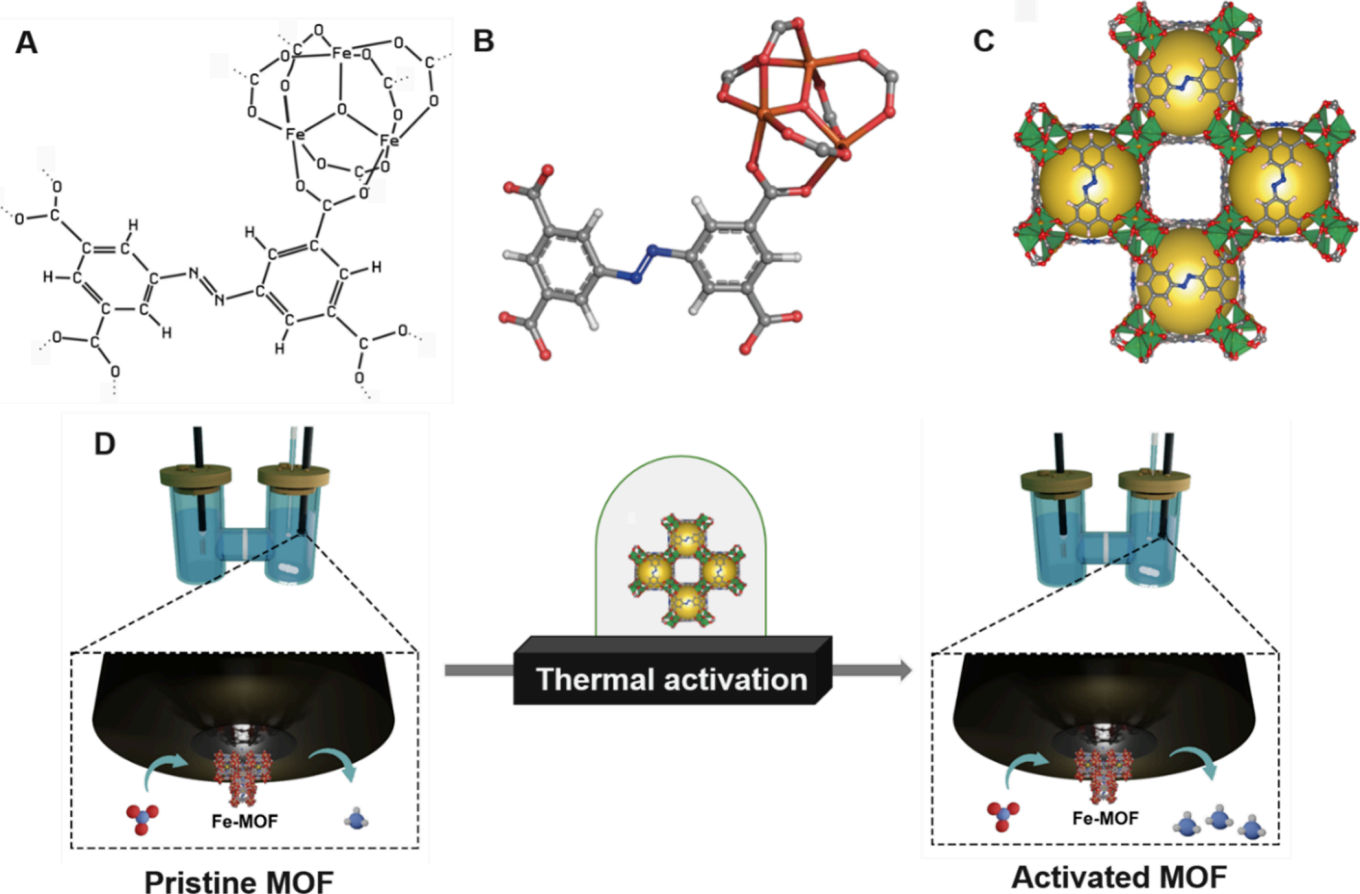
(A, B) Chemical Structure of the ABTC Linker with the Fe_3_-(μ_3_-O)(COO)_6_ Node. (C) Crystallographic Structure of the PCN-250-Fe_3_ MOF (Carbon Atoms Are Gray, Oxygen Red, Nitrogen Blue, Hydrogen White, and Iron Orange). (D) Schematic Representation of Thermal Activation of the MOF

Ground state structures of all investigated species were optimized by the M06-L method^44^ in combination with the Def2TZVP basis set^45^ utilizing the Gaussian 16 software.^46^ The M06-L functional is recommended for calculations of transition metal complexes and inorganic and organometallic systems.^44^ It displayed also a good performance in calculations of hydrocarbon adsorption on Fe-MOF-74^47^ and alkane oxidative dehydrogenation by Fe_2_Me MOF nodes.^48^ The spin-unrestricted formalism was applied in all calculations. The solvent effects were considered by applying the universal continuum model based on electron density.^49^ The computational hydrogen electrode method^50,51^ was applied to calculate reaction energies assuming that the chemical potential of electron−proton pair (μ_*H*^+^+*e^−^*_) is equal to the chemical potential of 1/2 H_2_ (μ_1/2*H*_2__). The structure of PCN-250 consists of trimetallic nodes bridged by ABTC linkers (ABTC = 3,3′,5,5′-azobenzenetetracarboxylate; Scheme 1A,B). A cluster model of the Fe_3_-(μ_3_-O)(COO)_6_ node with ABTC linkers replaced by formate ions was utilized in all calculations (Figure S4). This cluster model was found to give results with a sufficient accuracy.^48^ The trimetallic node consists either of three Fe(III) centers (Fe_3_(III)OH model; Figure S4A) or of two Fe(III) and one Fe(II) center (Fe_2_(III)Fe(II); Figure S4B).^48,52^ In the former case, to maintain neutrality of the network, counterions such as OH^–^ (considered here), F^–^, and Cl^–^ are usually added to one of the Fe(III) atoms. The spin multiplicity 16 of the Fe_3_(III) model and spin multiplicity 15 of the Fe_2_(III)Fe(II) model were considered according to ref (48).

## 3 Results and Discussion

### Pristine MOF toward Ammonia Synthesis

The PCN-250-Fe_3_ MOF was used as a base catalyst.^53−56^ This Fe-based MOF is formulated as an outcome of the reaction between precursor Fe_3_-μ_3_-oxo metal cluster and tetratopic azobenzene-based ABTC linkers (ABTC = 3,3′,5,5′-azobenzenetetracarboxylate). The orange crystals of the PCN-250-Fe_3_ MOF constitute three Fe(III) octahedra atoms that share one oxygen atom (μ_3_-oxo) with each other, subsequently connected by six ABTC ligands (Scheme 1A–C).

Scheme 1D showcases the overall outline of this study in employing Fe-based MOF electrocatalysts for the ammonia production. The morphology of the material was primarily assessed by SEM (Figure S1A), and EDS mapping confirms the presence of Fe, carbon (C), nitrogen (N), and oxygen (O) elements over the material surface, as shown in Figure S1B–E.

**Figure 1:**
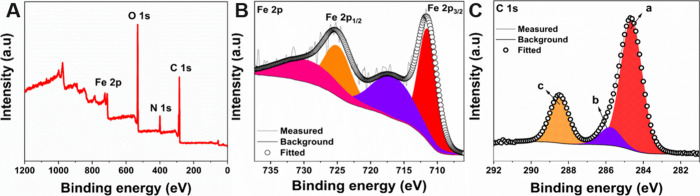
(A) XPS survey spectrum of the pristine PCN-250-Fe_3_ MOF phase. Deconvoluted XPS spectrum for (B) Fe 2p and (C) C 1s.

XPS was conducted on a pristine PCN-250-Fe_3_ MOF catalyst. The survey spectrum showed prominent sharp peaks of Fe 2p, C 1s, N 1s, and O 1s at their respective binding energies of 710, 284.6, 399.7, and 531.7 eV upon analysis from 0 to 1200 eV (Figure 1A). The quantitative analysis of the catalyst showed the presence of Fe 2p, C 1s, N 1s, and O 1s elements in the atomic percentage (at.%) of 2.14, 65.32, 6.09, and 26.45%, respectively. On deconvolution of the Fe 2p spectrum, the two peaks at 711.4 and 724.9 eV were obtained, which correspond to the Fe 2p_3/2_ and 2p_1/2_ states, respectively (Figure 1B). This clearly attributes to the +3 oxidation state of Fe centers in the PCN-250-Fe_3_ MOF catalyst. The satellite peaks of Fe 2p_3/2_ and 2p_1/2_ are also observed in the deconvoluted spectra at 716.6 and 729.4 eV.^57,58^ The C 1s spectra also gave three peaks at 284.6, 285.7, and 288.4 eV, which correspond to the C=O (peak a), C–N (peak b), and C=C/C–C (peak c), respectively (Figure 1C). The O 1s spectra also show the Fe–O (peak a) and O–H (peak b) bonding peaks as well shown in Figure S2. The XPS results of pristine/nonactivated MOF samples well match with literature data as well.^58^

**Figure 2:**
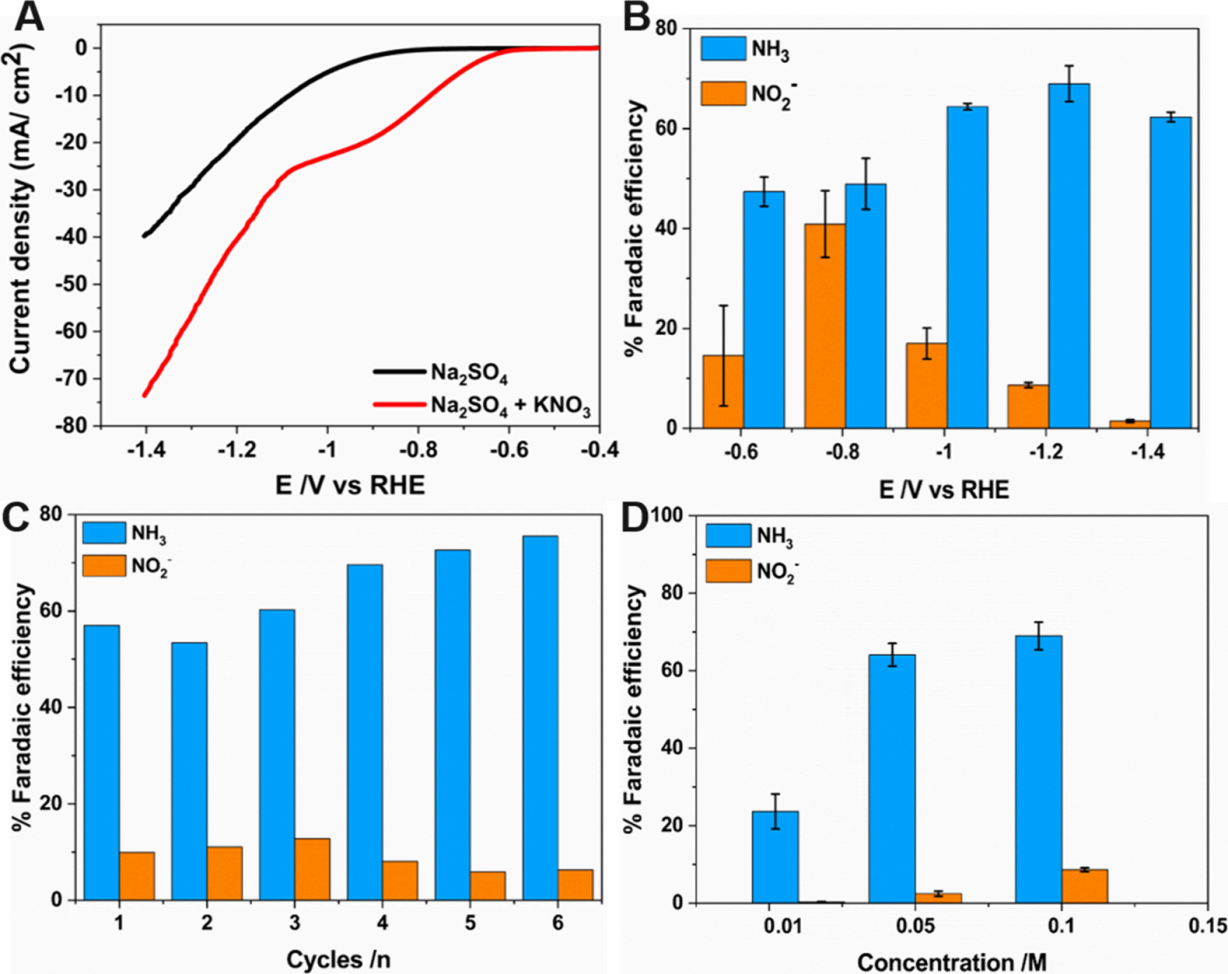
Electrocatalytic experiments of the pristine PCN-250-Fe_3_ MOF. (A) LSV curves in the Na_2_SO_4_ 0.5 M electrolyte with and without KNO_3_ 0.1 M. The experiment was conducted at a scan rate of 20 mV s^–1^. (B) Potential-dependent FE plots of NH_3_ and NO_2_^–^. (C) FE of NH_3_ and NO_2_^–^ on consecutive cycling electrolytic tests at −1.2 V vs RHE (the electrolyte used was Na_2_SO_4_ 0.5 M + KNO_3_ 0.1 M). (D) FEs of the nonactivated pristine Fe-based MOF sample toward NH_3_ and NO_2_^–^ at different concentrations of KNO_3_.

Electrocatalytic activity of the pristine PCN-250-Fe_3_ MOF toward NRA was primarily investigated using the linear sweep voltammetry (LSV) technique. The experiments were carried out with and without KNO_3_ in a 0.5 M Na_2_SO_4_ electrolyte. The electrocatalytic performance of these MOFs was studied in a three-electrode setup by drop-casting the MOF over GC, which served as the working electrode substrate. From the LSV curves, it is evident that the MOF electrocatalysts can reduce the NO_3_^–^ ions in the electrolyte, owing to the low onset potential and high current density, in comparison to electrolyte systems without NO_3_^–^ (Figure 2A). The current has been normalized with the geometrical surface area of the electrode. The area of the electrode was calculated using the equation Π*r*^2^, where *r* = 1.5 mm. The obtained current value via experimentation was divided by the area of the electrode surface. The major objective of the study is to track the behavior of the catalyst toward NRA via electrolysis in the proposed working potential range and to identify the peak potential that delivers the maximum FE in the procured volcano-shaped curve. The electrolysis measurements were executed in a H-type electrolytic cell with the cathodic and anodic compartments well separated by a frit. At the cathodic compartment of the cell, the electrocatalyst PCN-250-Fe_3_ MOF drop-casted over the GC serves as the working electrode, along with the reference electrode, while the counter electrodes were held at the anodic compartment. Each electrolytic experiment was carried out for 1 h at room temperature with continuous magnetic stirring (100 rpm) at the potential range previously mentioned. Post electrolysis, solution samples from the cathodic part were collected and quantified for ammonia and nitrite using the standard colorimetric method.^34,42,43^

Upon identifying the interesting behavior of the Fe-based MOF from the LSV measurements, NRA was performed sequentially via electrolysis in an H-type cell at potentials ranging from −0.6 to −1.4 V vs RHE. The concentration of NH_3_ and NO_2_^–^ was then analyzed using the colorimetric assay to calculate the FE of the catalyst toward these compounds. The results are plotted in Figure 2B. The nonactivated pristine MOF samples gave a maximum FE of 65% at −1.2 V vs RHE, and the FE was much lower at other measured potentials. Moreover, the stability of the electrocatalysts was assessed using six continuous chronoamperometry experiments for 1 h each, where no obvious decay in FE was seen (Figure 2C). Experiments were also repeated at different nitrate concentrations, using the nonactivated pristine MOF samples at −1.2 V vs RHE, as shown in Figure 2D. A decrease in FE was observed when the concentration of KNO_3_ reaches lower values (0.01 M), while 0.1 and 0.05 showcased a reasonable FE.

### Activated MOF toward Ammonia Synthesis

**Figure 3:**
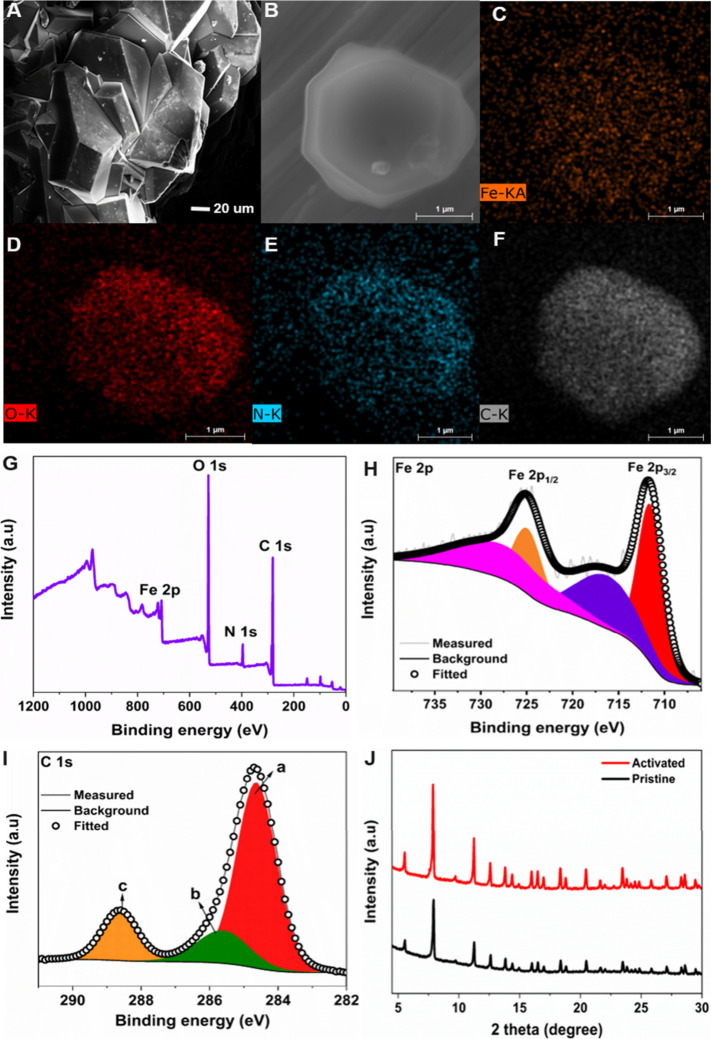
Activated PCN-250-Fe_3_ MOF (A, B) SEM image; (C–F) EDS mapping of elements (scale bar 1 μm); (G) XPS survey spectrum. Deconvoluted XPS spectrum of (H) Fe 2p and (I) C 1s; (J) XRD pattern of both activated and pristine MOF samples.

The MOFs were thermally activated in vacuum at 150 °C for 3 h and used for the subsequent characterization and electrocatalytic studies. Morphological and structural analysis of the activated PCN-250-Fe_3_ MOF material was carried out using SEM, EDS, XPS, and XRD studies and compared with the nonactivated pristine MOF to confirm if the structural integrity remains intact, as suggested by previous studies. The morphology of the material was primarily assessed by SEM, where dodecahedral-shaped crystals could be clearly identified, as shown in Figure 3A. A further magnified structure of the PCN-250-Fe_3_ MOF is given in Figure 3B. Elemental distribution over the sample surface was carried out by using EDS elemental mapping, where Fe, O, N, and C showed a uniform distribution over the sample surface (Figure 3C–F).

XPS was conducted for detailed elemental analysis of the activated PCN-250-Fe_3_ MOF for a better understanding of the electronic structure of the material and chemical states of the atoms in the material. The survey spectrum was measured for the elemental identification in the range of 0 to 1200 eV. Prominent sharp peaks of Fe 2p, C 1s, N 1s, and O 1s were observed at their respective binding energies, as shown in Figure 3G. The at. % Fe 2p, C 1s, N 1s, and O 1s elements were procured in the order of 3.93, 65.01, 5.06, and 26%, respectively. The deconvoluted Fe 2p spectrum displayed two peaks at 711.53 and 724.9 eV for Fe 2p_3/2_ and 2p_1/2_, respectively (Figure 3H). The satellite peaks are also evident in the deconvoluted Fe 2p spectra.^57,58^ The C 1s spectrum depicts three peaks at 284.6, 285.6, and 288.6 eV, which correspond to the C=O (peak a), C–N (peak b), and C=C/C–C (peak c), respectively (Figure 3I). The XPS results are in compliance with those previously reported in the literature.^58^ Further, XRD measurements were carried out for understanding the crystal structure and lattice arrangements of the material. The XRD patterns obtained from the activated PCN-250-Fe_3_ MOF material exhibited a crystalline nature, as shown in Figure 3J. The activated MOFs were further compared with the pristine form to identify if the former has undergone any possible alteration upon thermal activation. Based on this result, it is inferred that the activation has not brought any major changes to the material, in terms of its structural integrity, and has also retained the framework of the PCN-250-Fe_3_ MOF.^54,58,59^ Thus, the above characterization results showed that the structure of the material remains intact and is further evaluated by using the electrochemical experiments.

**Figure 4:**
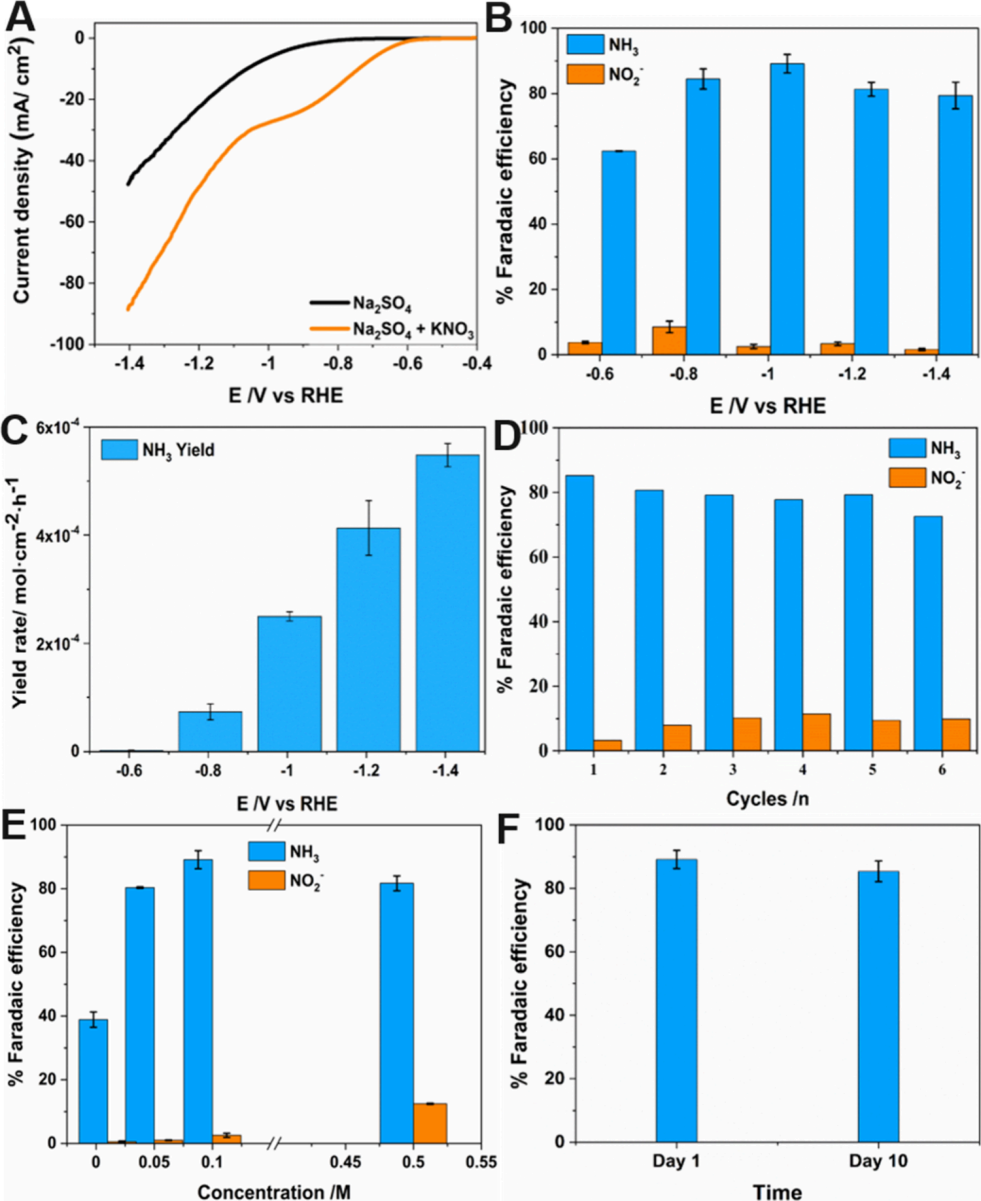
(A) LSV measurements of the activated PCN-250-Fe_3_ MOF in the Na_2_SO_4_ 0.5 M electrolyte with and without KNO_3_ 0.1 M. The experiment was conducted at a scan rate of 20 mV s^–^^1^. Electrocatalytic experiments of the activated PCN-250-Fe_3_ MOF (B) FE and (C) NH_3_ yield rate at various potentials. (D) FE of NH_3_ and NO_2_^–^ at consecutive cycling electrolytic tests at −1 V vs RHE (all electrolysis experiments from Figure 4B–D were carried out in the Na_2_SO_4_ 0.5 M + KNO_3_ 0.1 M electrolyte). (E) FEs of electrocatalysts toward NH_3_ and NO_2_^–^ at different concentrations of 0.01, 0.05, 0.1, and 0.5 M KNO_3_. (F) Stability assessments of activated MOF electrocatalysts toward NH_3_ production (FE) with time (electrolysis experiments carried out in the Na_2_SO_4_ 0.5 M + KNO_3_ 0.1 M electrolyte).

As in the case of the nonactivated pristine sample, the NRA of activated samples was preliminarily analyzed using the LSV technique. Figure 4A illustrates the electrochemical activity of the activated MOFs with and without nitrate in electrolyte systems containing 0.5 M Na_2_SO_4_. A difference in LSV measurements could be identified between the activated and pristine samples (Figure 2A), where the thermally activated samples exhibit a slightly lower overpotential over pristine samples. Further, we extend our studies toward NRA via a couple of experimental demonstrations to understand the performance of the activated PCN-250-Fe_3_ MOF electrocatalyst. This typically includes understanding the relevance of activation of the MOF prior to its use as an electrocatalyst for ammonia synthesis. Upon analysis, it was interesting to note that the activated catalyst delivered an FE of about 90% for ammonia at −1 V vs RHE and around 80% for ammonia production at the potential range between −0.8 and −1.4 V vs RHE, as shown in Figure 4B. Thus, the ability of the electrocatalyst to produce a reasonably high FE, via thermal activation, enables the potential of engineering and modifying multiple other MOF or similar materials for ammonia production. In addition, post electrolysis, morphological analysis of the activated Fe-based MOF catalyst was conducted to evaluate differences in the material structure. SEM and EDS mapping of the activated Fe-MOF electrocatalyst structure was carried out. The Fe-MOF catalyst retained its structure (Figure S3A) postcatalytic activity and further exhibited a uniform distribution of Fe, C, O, and N elements throughout the sample surface (Figure S3B–E). Figure 4C represents the yield rate, where a clear and proportional increase in the ammonia production with respect to the cathodic potential is observed, which is also expected.

Chronoamperometry tests were also conducted via continuous electrolysis cycles for 6 h at −1 V vs RHE to evaluate the stability of the electrocatalyst toward NRA. Ammonia and nitrite concentrations were measured, and FEs for all these samples were calculated. Figure 4D depicts the electrocatalytic stability of the PCN-250-Fe_3_ MOF electrocatalyst, where no obvious decay in FE after six cycles is seen. This further demonstrates the potential of the PCN-250-Fe_3_ MOF for real applications such as for NRA. The above results of the PCN-250-Fe_3_ MOF delivering maximum FE at −1 V vs RHE in 0.1 M KNO_3_ have prompted us to carry out experiments at various nitrate concentrations such as 0.5, 0.05, and 0.01 M to further evaluate the efficiency of the electrocatalyst toward NRA (Figure 4E). It was observed that the FE of the electrocatalyst at 0.5 and 0.05 M was slightly lower than 0.1 M; however, they are within the error range of FE at 0.1 M. Notably, at a higher concentration of nitrate (0.5 M), the FE of NO_2_^–^ is found to be high compared with 0.1 and 0.05 M. This could be possibly due to the fact that in concentrated nitrate solution, hydrogen adsorption is hindered^60^ due to excessive amounts of nitrate, resulting in an increased FE of NO_2_^–^ when compared to other systems. However, in the case of NH_3_, the FE remains almost the same or slightly decreased when compared to 0.1 M. On the other hand, a severe decline in FE for NH_3_ was obtained at 0.01 M, most probably due to the competitive adsorption of the supporting electrolyte (SO_4_^2–^). As described in the case of other metals, the mechanism of the reaction is influenced by the presence of strong adsorbates such as sulfate.^60^ When the nitrate concentration is 0.01 M or lower, sulfate acts as a competitive anion for active sites with a consequent decrease in FE for both NH_3_ and NO_2_^–^ at the optimal potential. This result accords with other observations in the literature.^40^ Furthermore, stability assessment of activated MOF samples was carried out by analyzing their FE toward ammonia production after storing them for 10 days under room temperature in aqueous media. No major difference in the FE of MOF electrocatalysts was observed during its storage for the ascribed duration (Figure 4F), confirming the stability of the activated sample.

### Evaluating Fe MOFs for Ammonia Production

The literature has often emphasized on the importance of activating MOFs via multiple techniques (conventional heating and vacuum, solvent-exchange, supercritical CO_2_ exchange, freeze-drying, chemical treatment, etc.) for their enhanced performance, owing to its increased porosity and surface area.^61−63^ Activation involves the removal of guest molecules such as volatile solvents or trapped entities from the MOF without affecting the structural integrity of the active material. Interestingly, the literature suggests that subjecting the Fe-MOF to varying pressures and temperatures can aid in enhancing its properties for targeted applications. A pressure-induced sequential phase transformation of the MOF was studied by Yuan et al., and the implications for MOF densification were analyzed, which showed a significant increase in the volumetric CH_4_ uptake (by 21%).^54^ Another interesting study was conducted by Day et al., where variation in activation temperature played a vital role in improving the MOF properties.^64^ In the above study, the thermal activation of the MOF carried out at 150 and 250 °C was employed toward acetylene adsorption. Notable observations put forward by the group include the removal of guest molecules such as volatile solvents from the MOF at 150 °C and decarboxylation of the ligand in open metal site formation at 250 °C, along with the formation of the mixed valent state of Fe(II/III) in the latter. This mixed valence can also aid in the enhancement of the gas adsorption performance. Hence, such investigations can play a vital role in improving the MOF properties and fabrication of application-specific catalysts.

**Figure 5:**
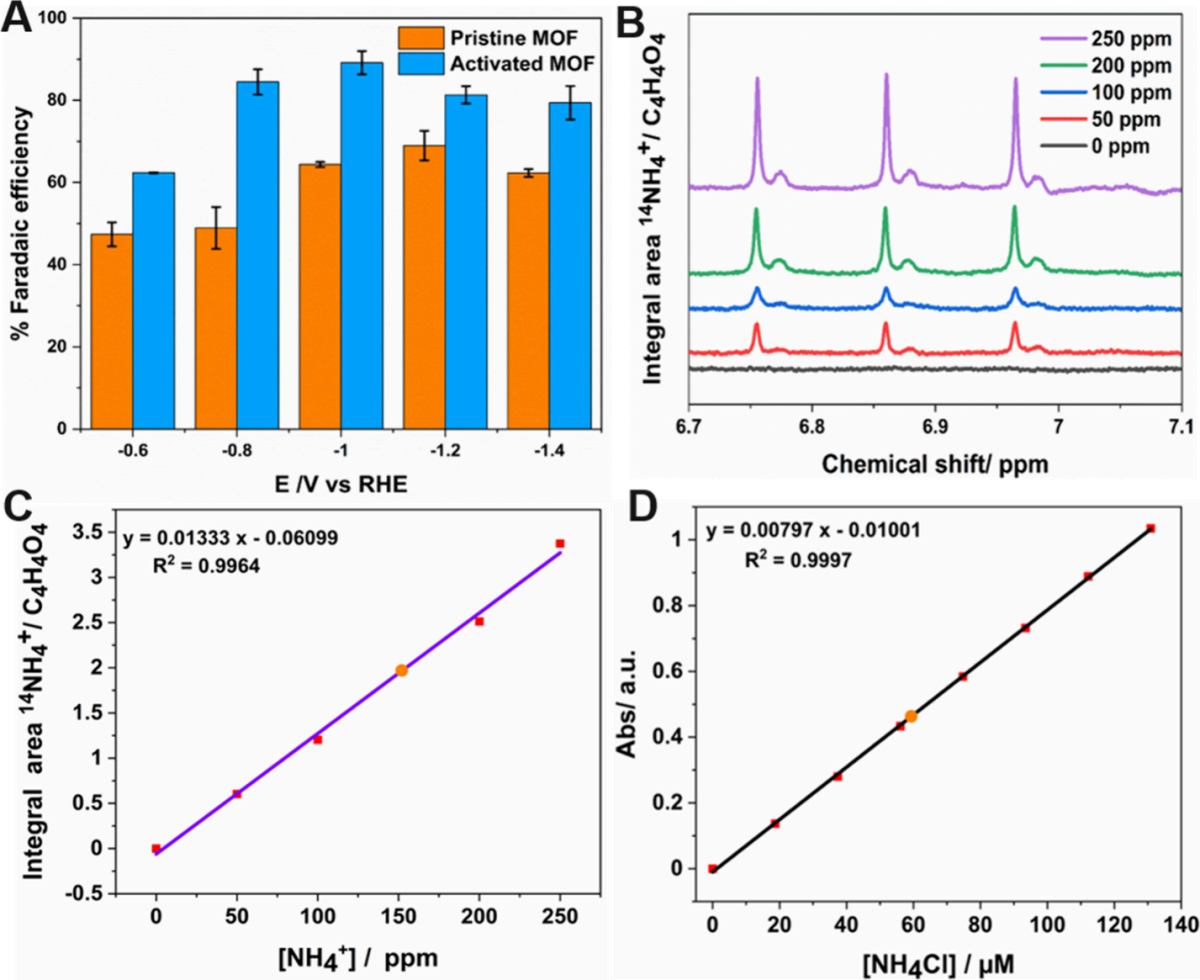
(A) Comparison of the FE of pristine and activated MOFs, respectively. (B) ^1^H NMR spectra at various concentrations corresponding to a calibration curve. (C) Calibration curve for ammonia determination using the ^1^H NMR method (the measured sample is depicted in orange). (D) UV calibration curve for ammonia determination using the colorimetric indophenol blue method (the measured sample is depicted in orange).

On analyzing the FE of MOF samples, a clear increase in FE was observed in activated samples, compared to the pristine nonactivated MOF, at every corresponding potential (Figure 5A). In other words, the nonactivated pristine MOF samples gave a maximum FE of 65% at −1.2 V vs RHE, while the potential of activated samples that delivered maximum FE was much lower (−1 V vs RHE) and delivered around 90% FE. Also, an evident increase in FE was observed at each corresponding potential of the activated MOF compared to the pristine samples. Thus, the observation on FE emphasizes the improvement in MOF properties upon activation, indicating this approach to be ideal for enhancing the material property. The possibility of enhancement in the PCN-250-Fe_3_ MOF via the mixed valence state of Fe(II/III) is expected at very high temperatures.^64^ Thus, without subjecting the Fe-MOF sample to a very high temperature of 250 °C, there was an increment in FE via the removal of volatile solvent molecules/guest molecules. In short, from an electrocatalytic point of view, the activation of samples is speculated to result in the exposure of more active Fe sites that are accessible for the nitrate ions in the electrolyte for ammonia production. The presence of the highly charged trivalent Fe(III) metal cation aids toward a strong metal–ligand coordination bond, eventually enhancing the thermostable properties of the Fe-MOF. With 650 °C known to be its decomposition temperature, the activation was employed only to 150 °C (around 1/4 of its decomposition temp), yet an enhancement in FE was evident in the studied MOF samples. Thermogravimetric studies on PCN-250-Fe_3_ MOF molecules also confirm that below 100 °C, the sharp weight loss could be attributed to the removal of surface-adsorbed water molecules,^65−67^ and/or subsequent heating, which aid toward removal of trapped entities, owing to the synthesis conditions. Thus, thermal activation is expected to remove the trapped entities on the pores, eventually exposing the catalytically active sites that are accessible for electrolyte ions for ammonia production. In principle, the activation of the MOF can modify the porosity of the material by increasing the active sites^68,69^ and thus creating more room for electrolyte accessibility for ammonia production.

Such activation-based approaches are evident toward carbon dioxide (CO_2_) based-reaction as well. For instance, some recent works emphasize the role of the microenvironments in the modulation of activity of different catalysts for relevant energy-related reactions such as CO_2_ reduction reaction.^70−73^ In fact, one of the factors relevant in the microenvironment’s modulation is the porosity of the material, wherein engineering the electrocatalyst structure appropriately can tailor the selectivity toward a specific C_1_ or C_2_ product.^71^ Likewise, the thermal activation of the MOF can enhance the efficiency of the material toward ammonia production. In short, although there are studies explaining the importance of activation, a better clarity can be brought via such experimental demonstrations that shall be helpful in understanding the potential of the electrocatalyst involved in the study. Carrying out such conclusive studies can also be beneficial in designing newer electrocatalysts by paying special attention to the careful activation of pristine materials for similar applications and/or for other interesting applications in the future. Furthermore, a comparison of various Fe-based catalyst systems has been detailed in Table S1, constituting their experimental conditions, FE, and yield rate in reducing nitrate to ammonia. To further confirm the reliability of colorimetric methods in determining the ammonia content, ^1^H NMR experiments were conducted. Figure 5B shows the ^1^H NMR spectra at various NH_4_Cl concentrations (ppm), delivering a typical triplet corresponding to the ^1^H NMR signal of the equivalent H in ^14^NH_4_^+^. Figure 5C depicts a calibration curve obtained by plotting the area of the ^1^H signal divided by the area of the signal corresponding to maleic acid (internal standard) as a function of the NH_4_Cl concentration. On comparing the calibration curve with the one procured using the colorimetric indophenol method (Figure 5D), a similar concentration value was obtained (59.52 μM for the colorimetric method and 58.72 μM for ^1^H NMR) with an error percentage of 1.4%. It was observed that the ammonia content, determined by two different methods, yielded a similar value (orange symbol in both calibration curves), providing additional confirmation of the accuracy of the colorimetric method used for ammonia quantification.

### Theoretical Studies

To obtain a deeper understanding of the NRA reaction mechanism catalyzed by the PCN-250-Fe_3_ MOF, step-by-step geometry optimizations of NO_3_^–^, NH_2_, and all intermediate species adsorbed on a cluster model of the PCN-250 catalyst were performed using DFT methods. Since the unit cell of PCN-250 consists of 416 atoms and calculations of the whole unit cell would be computationally ineffective, a cluster model of the Fe_3_-(μ3-O)(COO)_6_ node with ABTC linkers replaced by formate ions was utilized in all calculations (Figure S4).

The trimetallic node consisted of three Fe centers either all in oxidation state III (Fe_3_(III)OH model; Figure S4A) or two in oxidation state III and one in II (Fe_2_(III)Fe(II); Figure S4B) following the methodology of similar studies.^48,52^ In the former case, to maintain neutrality of the network, the OH^–^ counterion is usually placed close to one of the Fe(III) atoms.

**Figure 6:**
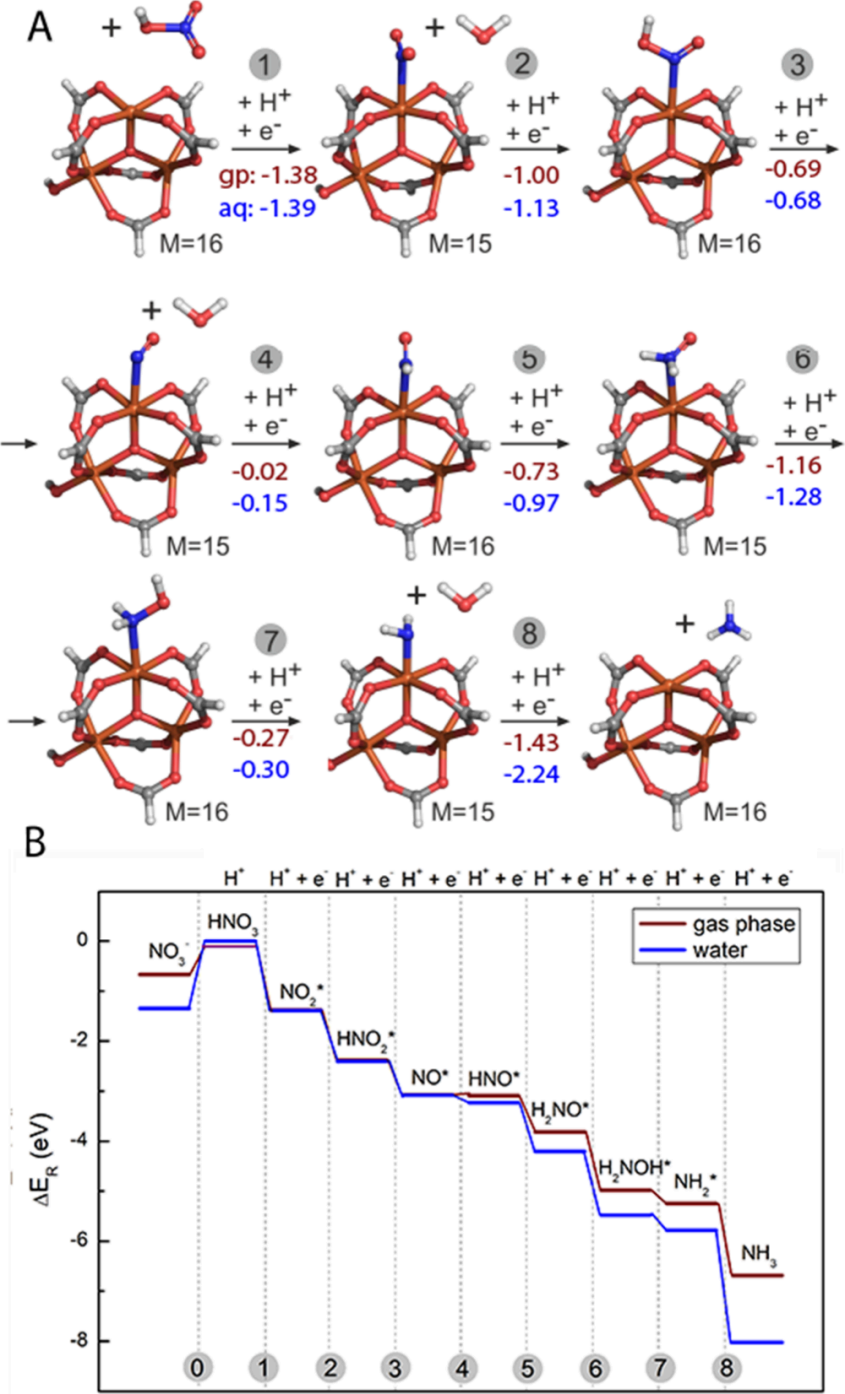
(A) Reaction mechanism of NRA catalyzed by PCN-250 (Fe_3_(III)OH model). The brown values are the reaction energies (Δ*E*_R_) in the gas phase, and the blue values are in water. The multiplicities (*M*) of all species are also reported. The carbon atoms are gray, oxygen red, nitrogen blue, iron orange, and hydrogen white. (B) Diagram of the NRA reaction energies for the reaction steps in the gas phase and water.

Using the Fe_3_(III)OH model, we have evaluated the energy profiles of different reaction routes that lead either to the formation of the desired NH_3_ or to the formation of other products (NO_2_, NO, N_2_O, N_2_, and HNO_2_; Figure S5). Considered reaction routes were based on the study of Wu et al.^34^ The most probable reaction mechanism of the NRA reaction (Figure 6) was calculated both in the gas phase and in the aqueous environment, which corresponded more to the used experimental conditions. It was initiated by a solution-mediated proton transfer to the NO_3_^–^ to form HNO_3_ (reaction energy Δ*E*_R_ = 0.67 eV in the gas phase and 1.35 eV in water; Figure 6B, step 0). The reaction then proceeded through eight-proton and eight-electron transfers (Figure 6A,B and Figure S6A,B, steps 1–8). Generally, all reaction steps were more favorable in the aqueous environment, which indicates that the presence of a solvent with a certain polarity facilitated the electron transfer and thus promoted the NRA reaction. It is worth noting that similar results were obtained by using the Fe_2_(III)Fe(II) model of the PCN-250 MOF (Figure S6) with one exception in step 4, which was slightly endothermic in the gas phase (Δ*E*_R_ = 0.27 eV). In short, these DFT results substantiate the feasibility of the NRA reaction mechanism of the studied reaction system.

## 4 Conclusions

In this study, we investigated the potential ability of PCN-250-Fe_3_ MOF electrocatalysts toward enhanced ammonia production via thermal activation. The activated MOF catalyst was successful in delivering a high FE of 90% at −1 V vs RHE, while pristine samples gave about 60% at the same potential. A clear enhancement in FE was observed for activated PCN-250-Fe_3_ MOF catalysts over the pristine material at every corresponding potential. The theoretical results are also in good accordance with the experimental results toward NRA by the PCN-250-Fe_3_ MOF electrocatalysts. Further, the stability of the activated material with time was also assessed for understanding the potential of the material. Thus, the above studies and observation can open up possibilities for researchers to tailor or surface engineer catalyst surfaces for fabricating the desired electrocatalyst for catalytic applications.

## Supporting Information Available

SEM and EDS mapping of the pristine PCN-250-Fe_3_ MOF sample, deconvoluted O 1s XPS spectra of the pristine MOF sample, SEM and EDS mapping of the Fe-MOF catalyst post catalysis, cluster models of the PCN-250-Fe_3_ MOF, and reaction pathways (PDF)
