# Supplementary material for: Fe-MOF Catalytic Nanoarchitectonic toward Electrochemical Ammonia Production
Source: ACS Appl Mater Interfaces. 2023 Oct 2;15(40):47294–306. doi: 10.1021/acsami.3c12822 (PMC10571008; doi:10.1021/acsami.3c12822)
Supplement: Supplementary file 1 — am3c12822_si_001.pdf [file am3c12822_si_001.pdf]

Supporting information

Fe-MOF Catalytic Nanoarchitectonic toward Electrochemical Ammonia Production

Akshay Kumar K. Padinjareveetil,[a] Juan V. Perales-Rondon,[a] Dagmar Zaoralová,[b] Michal Otyepka,[b,c] Osamah Alduhaish,[d] Martin Pumera[a,d,e f,g*]

[a] Future Energy and Innovation Laboratory, Central European Institute of Technology, Brno University of Technology, Purkyňova 123, 61200 Brno, Czech Republic

[b]IT4Innovations, VŠB – Technical University of Ostrava, 708 00 Ostrava-Poruba, Czech Republic

[c] Regional Centre of Advanced Technologies and Materials, Czech Advanced Technology and Research Institute (CATRIN), Palacký University Olomouc, 783 71 Olomouc, Czech Republic

[d] Chemistry Department P.O.Box 2455, College of Science King Saud University, Riyadh, 11451 Saudi Arabia

[e] Faculty of Electrical Engineering and Computer Science, VSB - Technical University of Ostrava, 17. listopadu 2172/15, 70800 Ostrava, Czech Republic

[f] Department of Paediatrics and Inherited Metabolic Disorders, First Faculty of Medicine, Charles University, Prague, Ke Karlovu 2, 128 08 Prague, Czech Republic

[g] Department of Medical Research, China Medical University Hospital, China Medical University, No. 91 Hsueh-Shih Road, Taichung 40402, Taiwan

* Author for correspondence; pumera.research@gmail.com

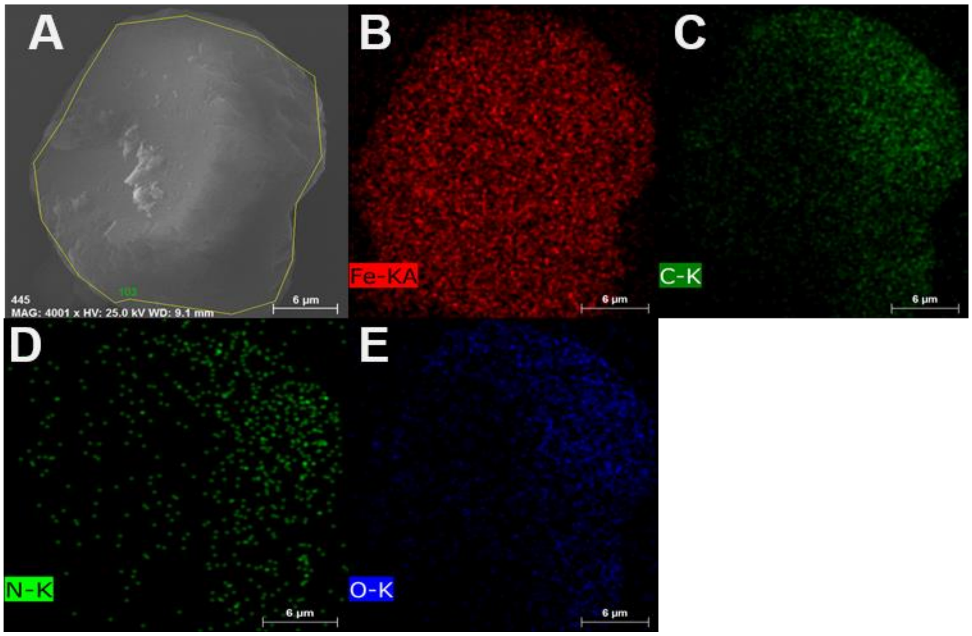

Figure S1. (A) SEM image of pristine PCN-250-Fe_3 MOF sample. (B-E) EDS mapping of elements.

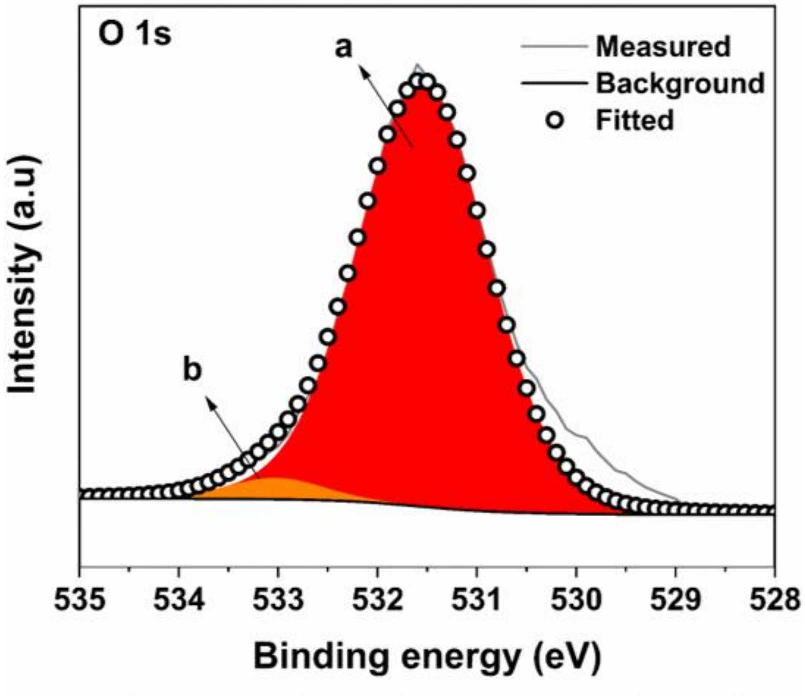

Figure S2. Deconvoluted O 1s XPS spectra of pristine PCN-250-Fe_3 MOF sample.

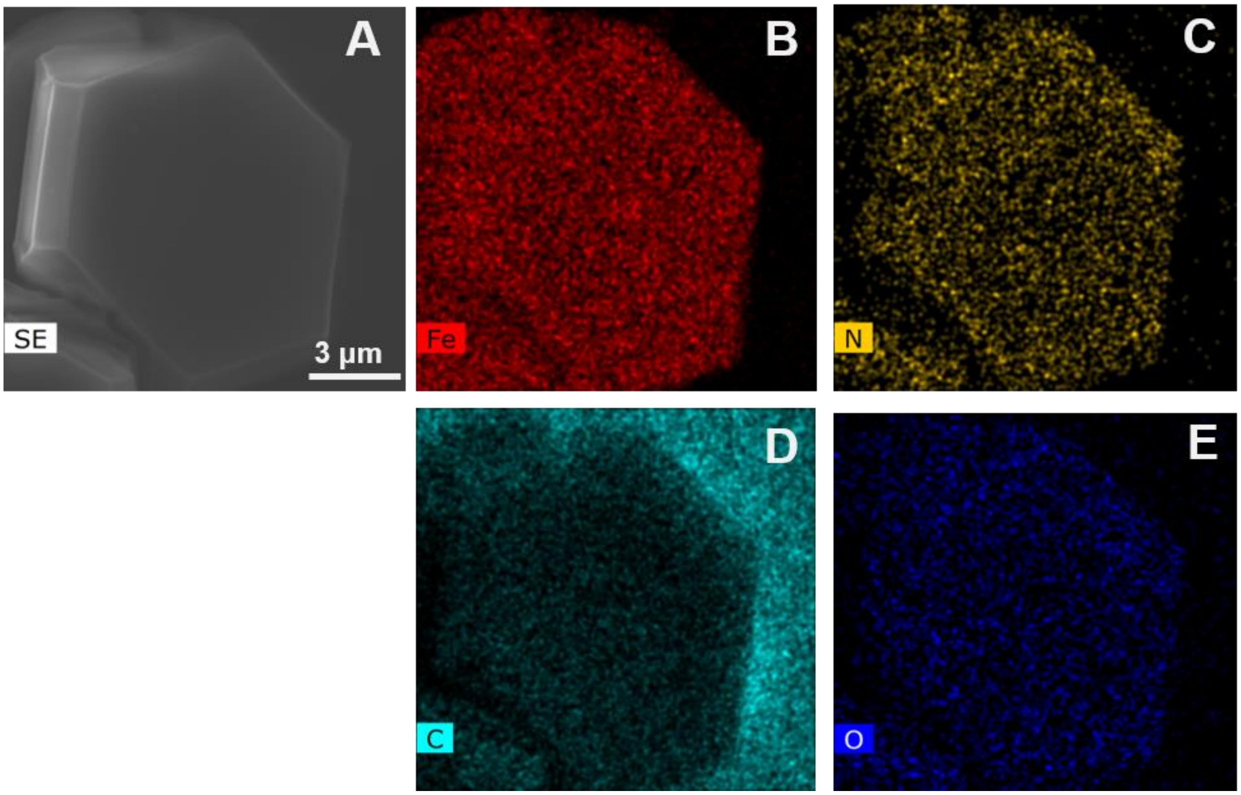

Figure S3. (A) SEM image of Fe MOF catalyst post electrocatalytic experiments. EDS mapping of (B) Fe, (C) N, (D) C, (E) O elements.

Table S1. Nitrate to ammonia conversion results using various Fe based catalysts.

Material	NH_3 FE / Potential	Yield rate	Electrolyte conditions	Reference
Fe single-atom catalysts (Fe-PPy SACs)	~100% at −0.7 V vs RHE	2.75 mg_{NH3} h^{-1} cm^{-2} at −0.7 V vs RHE	0.1 M KOH/ 0.1 M KNO_3	1
2D Fe-based cyano-coordination polymer nanosheets (Fe-cyano NSs)	~90.4% at −0.5 V vs RHE	42.1 mg $h^{-1}mg_{cat}^{-1}$ at −0.5 V vs RHE	1 M KOH/ 0.1 M KNO_3	2
Fe-doped Co_3O_4 nanoarray	95.5% at −0.7 V vs RHE	0.624 mg $mg_{cat.}^{-1}$ h^{-1} at −0.7 V vs RHE	0.1 M PBS/ 50 mM NO_3^-	3
Atomically dispersed FeMo-N-C SAC catalyst	94.7% at −0.45 V vs RHE	18.0 μmol cm^{-2} h^{-1}	0.05 M PBS/ 0.16 M KNO_3	4
Fe_1/NC at 900 ºC	86% at −0.7 V vs RHE	18.8 mg_{NH3} h^{-1} mg $_{cat}^{-1}$ at −0.9 V	0.1 M K_2SO_4 / 0.5 M KNO_3	5
Single-atom Fe-doped V_2O_5	97.1% at −0.7 V vs RHE	12.5 mg h^{-1} cm^{-2} at –0.7 V	1 M KOH/ 0.1 M KNO_3	6
Cu-Fe bimetallic catalysts (Cu_5Fe_5/OMC)	~70% at −0.5 V vs RHE	365.9 μg $h^{-1}mg_{cat}^{-1}$ at −0.8 V vs RHE	0.1 M PBS/ 500 ppm KNO_3	7
Fe single atom catalyst	~75% at −0.66 V vs RHE	~20,000 μg h^{-1} mg $cat._{}^{-1}$ at −0.85 V vs RHE	0.5 M KNO_3/ 0.10 M K_2SO_4	8
Co-doped Fe/Fe_2O_3	85.2 ± 0.6% at −0.75 V vs RHE	1,505.9 ± 130.5 μg h^{-1} cm^{-2} at −0.95 V vs. RHE	0.1 M Na_2SO_4 with $NaNO_3$	9
PCN-250-Fe_3 MOF (Activated)	~90% at − 1 V vs. RHE	2.5 x 10^{-4} mol cm^{-2} h^{-1} at −1 V vs. RHE	0.5 M Na_2SO_4 / 0.1 M KNO_3	This work

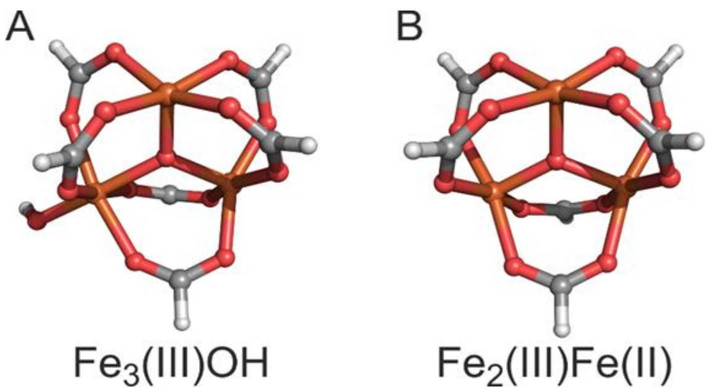

Figure S4. Cluster models of PCN-250. A) Model with all three Fe atoms in oxidation state +III. The OH^- group is added to maintain the system neutral. B) Model with two Fe atoms in oxidation state +III and one Fe atom in oxidation state +II. Carbon atoms are grey, oxygen red, iron orange and hydrogen white.

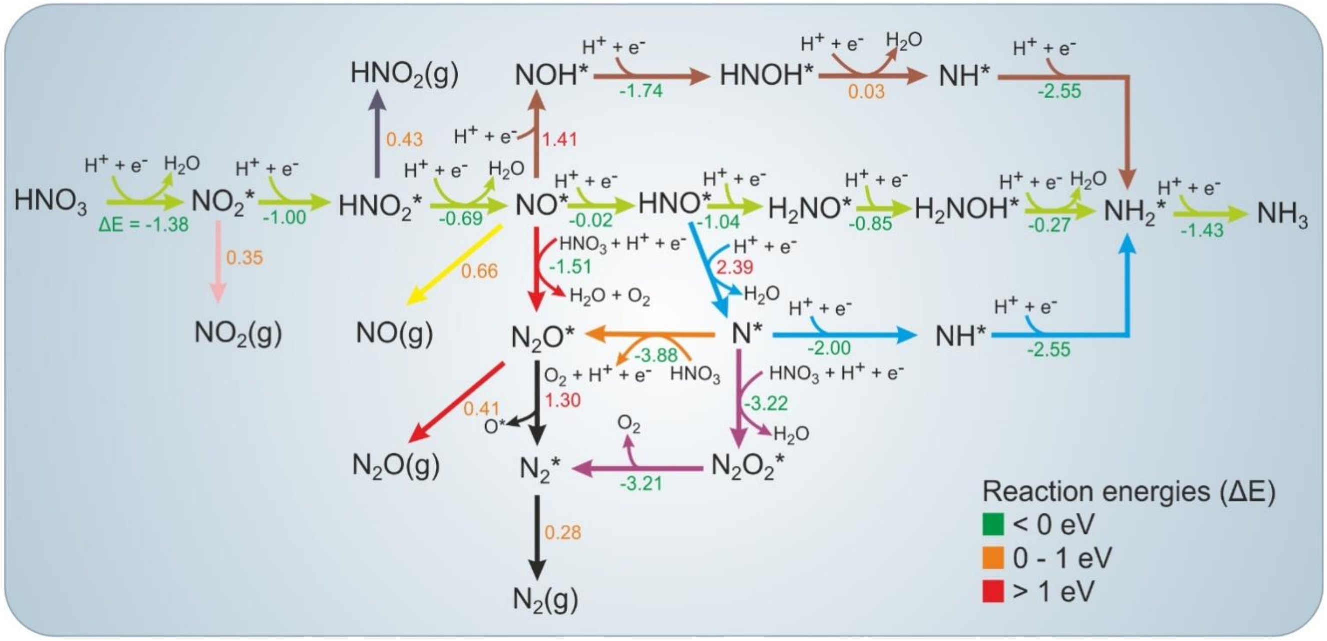

Figure S5. Possible reaction pathways of the NO_3^- reduction reaction based on the study of Wu et al.[34] catalyzed by PCN-250 represented as a Fe_3(III)OH model (Figure S4A) in the gas phase. Numbers under/next to arrows are reaction energies (ΔE) in eV. Reaction pathway marked by green arrows is depicted in Figure 6 and discussed in the main text.

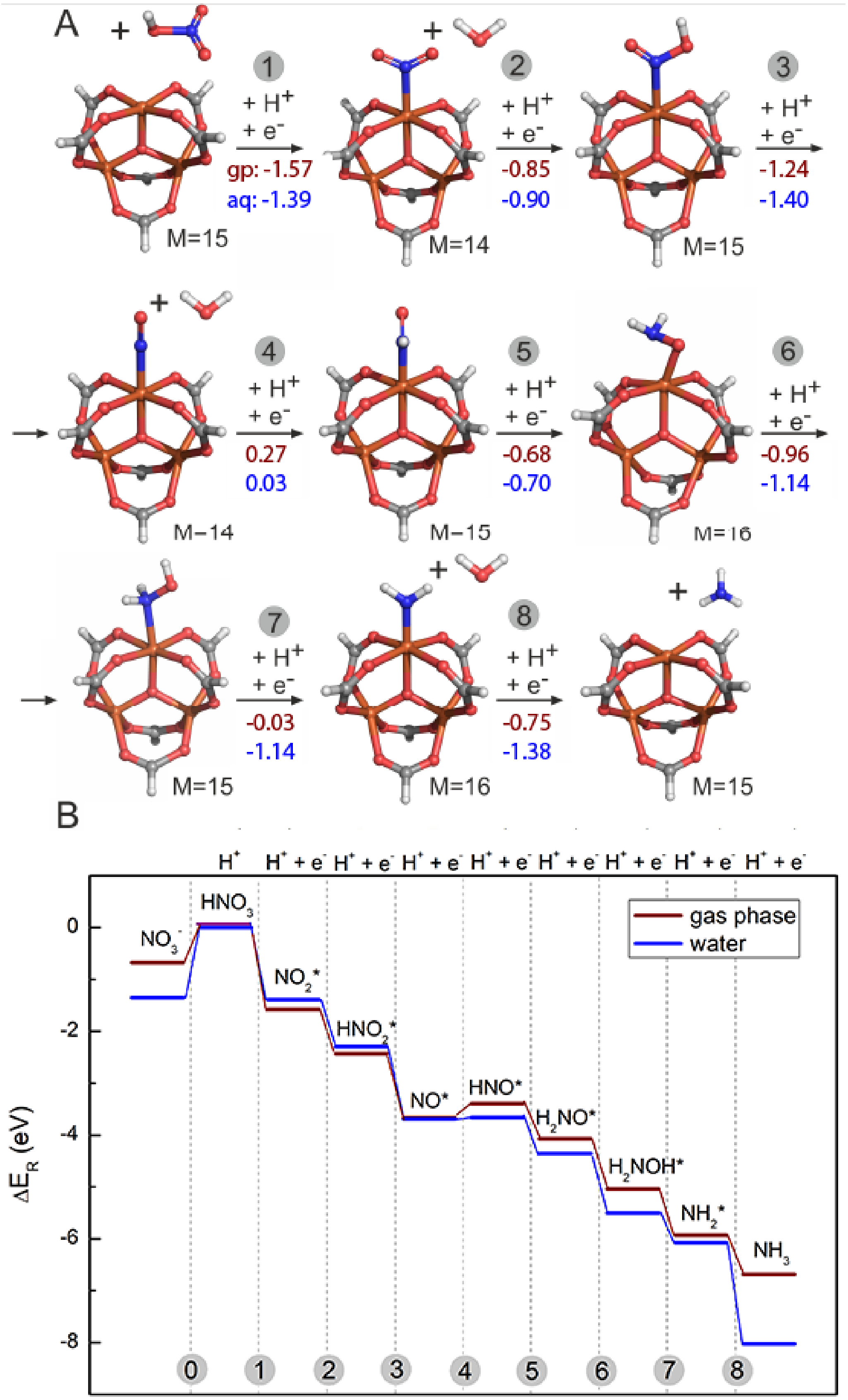
A
1
+ H+
+ e-
gp: -1.57
aq: -1.39
M=15
2
+ H+
+ e-
-0.85
-0.90
M=14
3
+ H+
+ e-
-1.24
-1.40
M=15
4
+ H+
+ e-
0.27
0.03
M=14
5
+ H+
+ e-
-0.68
-0.70
M=15
6
+ H+
+ e-
-0.96
-1.14
M=16
7
+ H+
+ e-
-0.03
-1.14
M=15
8
+ H+
+ e-
-0.75
-1.38
M=16
M=15
B
H+
H+ + e- H+ + e- H+ + e- H+ + e- H+ + e- H+ + e- H+ + e- H+ + e-
NO3-
HNO3
NO2*
HNO2*
NO*
HNO*
H2NO*
H2NOH*
NH2*
NH3
gas phase
water
ΔER (eV)
0
-2
-4
-6
-8
0 1 2 3 4 5 6 7 8

Figure S6. A) The reaction mechanism of the NRA catalyzed by the PCN-250 (Fe_2(III)Fe(II) model). Brown values are reaction energies (ΔE_R) in the gas phase, blue values in water. B) Diagram of the NRA reaction energies. The multiplicities (M) of species are also reported. Carbon atoms are grey, oxygen red, nitrogen blue, iron orange and hydrogen white.

References

(1) Li, P.; Jin, Z.; Fang, Z.; Yu, G. A Single-Site Iron Catalyst with Preoccupied Active Centers That Achieves Selective Ammonia Electrosynthesis from Nitrate. *Energy Environ. Sci.***2021**, *14* (6), 3522–3531. https://doi.org/10.1039/d1ee00545f.

(2) Fang, Z.; Jin, Z.; Tang, S.; Li, P.; Wu, P.; Yu, G. Porous Two-Dimensional Iron-Cyano Nanosheets for High-Rate Electrochemical Nitrate Reduction. *ACS Nano***2022**, *16* (1), 1072–1081. https://doi.org/10.1021/acsnano.1c08814.

(3) Wei, P.; Liang, J.; Liu, Q.; Xie, L.; Tong, X.; Ren, Y.; Li, T.; Luo, Y.; Li, N.; Tang, B.; et al. Iron-Doped Cobalt Oxide Nanoarray for Efficient Electrocatalytic Nitrate-to-Ammonia Conversion. *J. Colloid Interface Sci.***2022**, *615* (February), 636–642. https://doi.org/10.1016/j.jcis.2022.01.186.

(4) Murphy, E.; Liu, Y.; Matanovic, I.; Guo, S.; Tieu, P.; Huang, Y.; Ly, A.; Das, S.; Zenyuk, I.; Pan, X.; et al. Highly Durable and Selective Fe- and Mo-Based Atomically Dispersed Electrocatalysts for Nitrate Reduction to Ammonia via Distinct and Synergized NO2- Pathways. *ACS Catal.***2022**, *12* (11), 6651–6662. https://doi.org/10.1021/acscatal.2c01367.

(5) Liu, L.; Xiao, T.; Fu, H.; Chen, Z.; Qu, X.; Zheng, S. Construction and Identification of Highly Active Single-Atom Fe1-NC Catalytic Site for Electrocatalytic Nitrate Reduction. *Appl.*

*Catal. B Environ.***2023**, *323*, 122181. https://doi.org/10.1016/j.apcatb.2022.122181.

(6) Zhang, N.; Zhang, G.; Shen, P.; Zhang, H.; Ma, D.; Chu, K. Lewis Acid Fe-V Pairs Promote Nitrate Electroreduction to Ammonia. *Adv. Funct. Mater.***2023**. https://doi.org/10.1002/adfm.202211537.

(7) Zhao, J.; Liu, L.; Yang, Y.; Liu, D.; Peng, X.; Liang, S.; Jiang, L. Insights into Electrocatalytic Nitrate Reduction to Ammonia via Cu-Based Bimetallic Catalysts. *ACS Sustain. Chem. Eng.***2023**, *11* (6), 2468–2475. https://doi.org/10.1021/acssuschemeng.2c06498.

(8) Wu, Z. Y.; Karamad, M.; Yong, X.; Huang, Q.; Cullen, D. A.; Zhu, P.; Xia, C.; Xiao, Q.; Shakouri, M.; Chen, F. Y.; et al. Electrochemical Ammonia Synthesis via Nitrate Reduction on Fe Single Atom Catalyst. *Nat. Commun.***2021**, *12* (1), 1–10. https://doi.org/10.1038/s41467-021-23115-x.

(9) Zhang, S.; Li, M.; Li, J.; Song, Q.; Liu, X. High-Ammonia Selective Metal-Organic Framework-Derived Co-Doped Fe/Fe2O3 Catalysts for Electrochemical Nitrate Reduction. *Proc. Natl. Acad. Sci. U. S. A.***2022**, *119* (6). https://doi.org/10.1073/pnas.2115504119.
